# An oversampling method for imbalanced data based on spatial distribution of minority samples SD-KMSMOTE

**DOI:** 10.1038/s41598-022-21046-1

**Published:** 2022-10-07

**Authors:** Wensheng Yang, Chengsheng Pan, Yanyan Zhang

**Affiliations:** Intelligent Network and Information System, School of Electronic & Information Engineering, Nanjing University of Information Science & Technology, Nanjing, 210044 China

**Keywords:** Computer science, Scientific data

## Abstract

With the rapid expansion of data, the problem of data imbalance has become increasingly prominent in the fields of medical treatment, finance, network, etc. And it is typically solved using the oversampling method. However, most existing oversampling methods randomly sample or sample only for a particular area, which affects the classification results. To solve the above limitations, this study proposes an imbalanced data oversampling method, SD-KMSMOTE, based on the spatial distribution of minority samples. A filter noise pre-treatment is added, the category information of the near-neighbouring samples is considered, and the existing minority class sample noise is removed. These conditions lead to the design of a new sample synthesis method, and the rules for calculating the weight values are constructed on this basis. The spatial distribution of minority class samples is considered comprehensively; they are clustered, and the sub-clusters that contain useful information are assigned larger weight values and more synthetic sample numbers. The experimental results show that the experimental results outperform existing methods in terms of precision, recall, F1 score, G-mean, and area under the curve values when the proposed method is used to expand the imbalanced dataset in the field of medicine and other fields.

## Introduction

Imbalanced learning is a basic problem in machine learning. When the number of samples from different categories in a classification task dataset differs significantly, the dataset is called imbalanced. Typically, the category with the larger number of samples is called the majority category, and that with the smaller number of samples is called the minority category. Imbalanced data are widely available in various real fields, such as financial fraud detection^1^, medical disease diagnosis^2,3^, network intrusion detection^4,5^, smart homes^6^ and network fault diagnosis^7^. Minority categories in these fields have smaller sample sizes and poorer sample data quality; however, they typically carry more important information. Thus, we focus on a model’s ability to correctly classify the minority classes of samples, such as a complex network system, where it is more important to accurately diagnose the network fault types and maintain the normal operation of the system than to diagnose the network as normal.

However, in the classification process, traditional classification methods, such as support vector machines, decision trees, Bayesian networks, and k-nearest neighbours, are designed to maximise the overall classification accuracy. This often leads to higher classification accuracy for the majority category and lower classification accuracy for the minority category, which may be limiting for practical applications. The methods used to solve the imbalanced data problem and improve classification accuracy for minority class samples are divided into two main aspects. The first scheme is primarily from the perspective of the data. The main method comprises sampling, sampling through a strategy, and changing the category of the sample distribution to achieve the purpose of balancing the majority and minority class samples. There are three types of sampling strategies: undersampling^8,9^, oversampling^10,11^, and mixed sampling^12^. The second scheme is primarily from the perspective of the algorithms. By analysing the variability of the algorithm cost in different misclassification cases, the classification algorithm is optimised to improve the performance of the learning algorithm^13^. Data-based approaches are more popular in existing literature than approaches that improve a specific classification algorithm for a specific imbalanced dataset.

Among the three sampling methods, oversampling is used more frequently than the other two. Undersampling reduces the number of samples, which may lead to the loss of important data information. In addition, Batista et al. demonstrated that oversampling generally performs better than undersampling, as measured by the area under the ROC curve (AUC)^14^. The synthetic minority oversampling technique (SMOTE) is considered the most influential data pre-processing and sampling algorithm^15,16^. The basic idea of the SMOTE algorithm is to analyse the minority class samples, and based on them, manually synthesise new samples to add to the dataset^17^. SMOTE, as an undersampling method, balances the proportion of sample classes in the dataset to some extent by increasing the number of minority class samples, thereby improving the classifier's ability to classify minority classes while leaving its ability to classify majority classes unchanged^18^.

Although SMOTE is favoured in existing literature for its ability to solve imbalanced data problems and its implementational simplicity, it still has some limitations. SMOTE treats all minority class samples equally and does not consider the class information of the neighbouring samples. This often leads to the phenomenon of sample aliasing, which results in a poor classification effect. In addition, if the selected minority class sample is surrounded by minority class samples, the newly synthesised sample will not provide substantial useful information. If the selected minority class samples are surrounded by majority class samples, such samples may be noise; therefore, the newly synthesised samples will overlap more with the surrounding majority class samples, thereby resulting in classification difficulties.

In response to the above limitations of SMOTE, this study proposes an imbalanced data oversampling method, SD-KMSMOTE, based on the spatial distribution of minority samples. In contrast to SMOTE, SD-KMSMOTE considers the distribution of majority class samples around minority class samples. In addition, SD-KMSMOTE no longer treats all minority class samples equally, and completes the synthesis of new synthetic samples by considering the spatial distribution structure of minority samples. Specifically, the main contributions of this study are as follows:

1. Increasing the filtering noise pre-treatment. Before the new sample synthesis operation, the isolated minority class sample points that are surrounded by majority class samples are screened out as noise. Thus, overlap between the newly synthesised samples and majority class samples is avoided, and the differentiability between sample classes is enhanced.
2. Design of a new sample synthesis method. In order to solve the problem of how to no longer treat all minority class samples equally, this study divides minority class samples into several regions, and allocates different numbers of synthetic samples according to the sample clusters in different regions to solve this problem. Specifically, K sub-clusters are generated for a small number of classes of samples using the k-means method. With the total number of newly synthesised samples constant, the cluster is considered a unit, and the sub-clusters that contain more useful information are assigned larger weight values and a greater number of synthesised samples. This method comprehensively considers the spatial distribution of minority samples, allocates different numbers of composite samples to sample clusters in different regions, optimises the spatial distribution structure of new synthetic samples, and thereby improves the classification ability of the classifier for minority class samples.
3. Construction of calculation rules for the weight values. In order to solve the problem of how to use the useful information contained in the sample clusters in different regions to realize the synthesis of new samples in different regions. This study solves this problem by constructing a calculation rule for the weight value. Specifically, by calculating the Euclidean distance from the cluster centre of each cluster to the class centre of the majority class sample, the clusters that are closer to the majority class sample are assigned a higher weight value. Samples at boundary locations are more likely to be misclassified in the actual modelling process; therefore, using the sample information at boundary locations to generate new samples can give the model a larger sign and provide sufficient information for classification, which can effectively reduce the false positive rate of the classifier for the majority class sample.

## Related work

The imbalanced data problem is a research hotspot in the field of machine learning. Recently, an increasing number of studies have attempted to address this problem. The section only summarises the SMOTE-based improvement and extension methods in oversampling techniques.

### Filter the sample noise

One of the SMOTE noise filtering operations is denoising at the end of the SMOTE process, and two typical techniques for this are SMOTE-TomekLinks and SMOTE + ENN^19^, where the noise generated by newly synthesised samples can also be filtered. Both methods have been applied in various scenarios^20,21^. Another noise-filtering operation is to enhance SMOTE by adding filters. Puntumapon and Waiyamai proposed an algorithm, TRIM, to solve the problem of the over-generalization of SMOTE. That is, the synthetic data is generated to majority class regions, and the purpose of removing noise from minority class regions is achieved by iteratively filtering irrelevant majority class data from the exact minority class regions^22^. However, the method only targets minority class samples; therefore, the following situation may arise. The algorithm searches the minority class region located at the boundary; if the number of minority class samples is significantly less than the number of majority class samples, that is, the minority class samples are the noise at this time, the majority class samples are still filtered out as noise. This will erroneously remove useful information from the boundary region and blur the demarcation line, which in turn affects the classification performance of the data as a whole (including minority class samples and majority class samples). In addition, other methods to enhance SMOTE by adding filters include rough set-based filtering^23,24^ and ensemble-based filtering^25^. In summary, although many studies have explored the filtering of sample noise, implementing this for SMOTE still requires further research.

### Based on initial sample selection

Unlike SMOTE, which randomly selects objects to synthesize new samples, the proposed method is based on the initial sample selection. Typically, the objects for the best synthesised samples need to be selected by a method before the interpolation operation. This strategy aims to reduce overlap and noise in the final dataset. Based on SMOTE, Han and Wang designed two new minority class-based oversampling methods, Borderline-SMOTE1 and Borderline-SMOTE2^26^. Ref.^26^ considered that samples on the boundary (Danger) are more likely to be misclassified than samples far from the boundary (Safe); thus, boundary samples are more important for classification. Therefore, the algorithm improves the class distribution of samples by using only minority-class samples on the boundary to synthesise new samples. Abdi et al. proposed an oversampling method based on the Marcian distance to synthesise samples in only minority class-dense regions, which can effectively overcome the sample overlap problem; however, this method cannot guarantee the boundary sample information of minority classes^27^. Both of the two abovementioned methods one-sidedly synthesise new samples in a specific region of the minority class without considering the general influence of data from other regions. In addition, if there are few samples in the boundary region, it will be difficult to select candidate samples for the synthesis of new samples. In Refs.^28^ and^29^, the initial selection of samples was performed using a machine learning approach. In Ref.^28^**,** initial points were selected in support vectors that were obtained using support vector machines. In^29^, an adaptive semi-unsupervised weighted oversampling method was proposed using a semi-unsupervised hierarchical clustering approach, which implements the sampling of hard-to-learn instances near decision boundaries. The two abovementioned methods provide new ideas on how to select candidate interpolation samples.

### Sample-based adaptive generation

He et al. proposed a new adaptive synthetic sampling method (ADASYN) for the learning of imbalanced datasets. The basic idea of ADASYN is to use weighted distributions for different minority classes of samples based on their difficulty of learning; this generates more synthetic data for the minority samples that are more difficult to learn^30^. This ADASYN learning mechanism has led to a wide range of applications in different fields^31,32^.

In summary, although the abovementioned improved oversampling method based on SMOTE enables the synthesis of new samples and achieves good classification results in some specific cases, there are still some associated limitation. The spatial distribution structure of minority samples is not considered comprehensively, and only a single new sample is synthesised for a specific region, thereby neglecting some minority samples that contain important information. The method has no ability to mitigate intra-class and inter-class distribution imbalances while avoiding noise generation. No time or space complexity is considered; however, the respective improvements are achieved at the cost of high complexity, which makes it difficult to apply them in practice. To this end, this study proposes a new version of SMOTE based on the spatial distribution of minority class samples; it divides the minority class samples into several clusters by clustering and filtering the isolated sample points that are not in the clusters or are far from the clusters as noise. According to the distance between each cluster and the majority of samples, weight is assigned to each cluster. The number of new samples synthesised in different proportions is allocated according to the weight given to each cluster. Unlike Ref.^26^, which only targets the boundary region for new sample synthesis, this study comprehensively considers the spatial distribution of minority class samples and removes noise without destroying the intra-class and inter-class distributions. Moreover, the computational complexity is low, which can be directly applied to most classifiers to further improve the classification ability of the sample dataset as a whole with the premise of improving the classification accuracy of minority class samples.

## Methods

### SD-KMSMOTE

According to the definition in Ref.^26^, a minority class of samples is classified into three categories during the sampling process: safe, dangerous, and noise. When more than half of the samples around a minority class are minority class samples, the sample is considered to be in a safe region; when more than half of the samples around a sample are majority class samples, the sample is considered to be in a border (dangerous) region; and when all samples around a sample are majority class samples, the sample is considered noise. Because the study in Ref.^26^ considered that minority classes of samples in the boundary region contain the most useful information, only samples from that region are sampled, and new samples are synthesised. However, the boundary region contains the most important samples that are beneficial for classification, which does not imply that there are no important samples in other regions.

Therefore, inspired by Ref.^26^, this study proposes an imbalanced data oversampling method, SD-KMSMOTE, based on the spatial distribution of minority samples. The basic idea of SD-KMSMOTE is that, unlike Ref.^26^ which only samples samples from edge regions, in this study, by comprehensively consider the spatial distribution of minority class samples by utilizing ideas from Ref.^26^ and assigning more synthetic new samples in the boundary region, while also assigning a particular number of new samples to be generated in the safe region, And by designing a new sample synthesis method and constructing the calculation rules of the weight value, the allocation of the number of new samples to be synthesized in different regions is completed.

**Figure 1:**
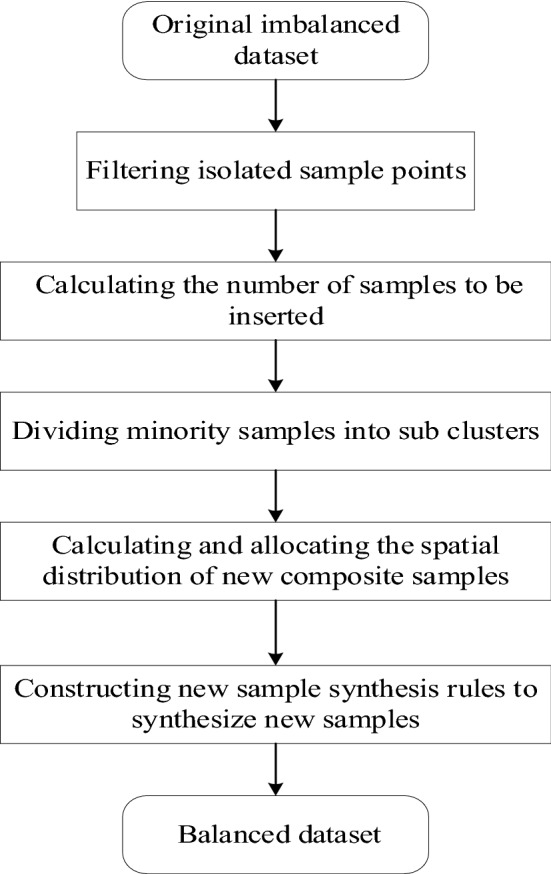
Flow chart of SD-KMSMOTE method.

SD-KMSMOTE is specifically implemented by introducing the k-means method to generate K sub-clusters by clustering minority class samples, thereby removing isolated points (noise) away from or not in the clusters before oversamling. The cluster is considered a unit, and different weight values are assigned to each cluster. With a constant total number of newly synthesised samples, clusters with higher weight values are assigned a higher number of synthetic samples. The calculation rules for weight values are constructed by calculating the Euclidean distance from the cluster centre of each cluster to the class centre of the majority class sample; the clusters that are closer to the majority class sample are assigned a higher weight value. The flow chart on the SD-KMSMOTE oversampling method is shown in Fig. 1, and detailed steps are presented below.

Let the original training sample be $S$ with number $s$, the minority class sample be $L$ with number $l$, and the majority class sample be $M$ with number $m$. Then, the equilibrium rate $E$ of sample $S$ is:

1 $$E = \frac{l}{m}$$

The closer E is to 1, the closer the number of majority class and minority class samples in sample S.

1. Filtering isolated sample points According to the K-neighbor algorithm, the k-neighbourhood of all sample points in sample $L$ is calculated. The Euclidean distance between the two sample points is calculated by the Euclidean distance formula (Formula 5). If there is a sample point for which the k-neighbourhood points are all majority class samples, it is considered an isolated sample point and the deleted, where the number of isolated sample points is ${l}_{outlier}\left({l}_{outlier}\ge 0\right)$.
2. Calculating the number of samples to be inserted. The balance rate $E^\text{'}$ of the samples is set after sampling, and the number of minority classes samples is calculated after sampling $l^\text{'} = E^\text{'}M$, then the number of samples to be inserted ${l}_{add}$ is:
  2 $$l_{add} = l^\text{'} - \left( {l - l_{outlier} } \right).$$
3. Calculating the Euclidean distances ${\mathrm{d}}_{1}, {\mathrm{d}}_{2},{\mathrm{ d}}_{3}, \dots ,{\mathrm{d}}_{\mathrm{K}}$ from the cluster centre of each cluster to the M-class centre of the majority class sample.
  Among them, in order to avoid differences in clustering results caused by the selection of initial clustering centers. We use the K-means +  + algorithm to improve the initial clustering center selection part of the K-means algorithm. The initial clustering center selection idea of the improved algorithm is as follows: The distance between the initial clustering centers should be as far as possible; Assuming that n initial cluster centers have been selected (n < k), when selecting the n + 1st cluster center, the farther away from the current n cluster centers, the higher the probability of being selected as the n + 1st cluster center.
  In addition, in order to avoid the k-means method falling into local optimization, we use the sum of the squared errors (SSE) to select the K value:
  3 $$SSE = \mathop \sum \limits_{i = 1}^{k} \mathop \sum \limits_{{p \in C_{k} }} \left| {p - m_{k} } \right|^{2}$$

In the formula, ${C}_{k}$ denotes the k-th cluster, P denotes the sample point in ${C}_{k}$, ${m}_{k}$ denotes the center point of K clustering centers, SSE denotes the clustering error of all samples, the smaller SSE is, the higher the sample aggregation degree is. When k is less than the true clustering number, due to the increase of k will greatly increase the clustering degree of each cluster, so the SSE drop will increase, and when k arrived true clustering number, the return of clustering degree obtained by increasing K will decrease rapidly, so the decline of SSE will decrease sharply, and then tend to be flat with the continuous increase of K value. The first point where it flattens out is the right K.

(3.1) The class centre of class M is calculated. The class centre of sample M defined in this study is obtained by finding the mean of all sample points in the class using the following method:
  Let there be $i\left(i=1, 2, 3, \dots , n\right)$ features in $m$ samples in class $M$, and ${x}_{i}$ denotes the i-th feature of the sample; for example, when $i=1$, that is, ${x}_{1}$ denotes the first feature of the sample, then the average value of the i-th feature of the sample in class ${A }_{{x}_{i}}$ is
  4 $$A _{{x_{i} }} = \frac{1}{m}\mathop \sum \limits_{j = 1}^{n} x_{ij} .$$
  In the formula, ${x}_{ij}$ denotes the value of the i-th feature in the j-th sample, for example, ${x}_{11}$ denotes the value of the first feature in the first sample of class $M$. The mean value of all samples in each feature in class $M$ is calculated separately according to the above formula, and the class centre sample $\overline{M }$ of class $M$ is $\left({A }_{{x}_{1}},{ A }_{{x}_{2}}, {A }_{{x}_{3}}, \dots , {A }_{{x}_{n}}\right)$.

(3.2) The distance between each cluster and M-class is calculated. Let the cluster centre of K subclusters obtained using the k-means method be ${C}_{i}(i=1, 2, 3, \dots ,K)$, then ${C}_{i}=\left({{C}_{i}}_{{x}_{1}},{ {C}_{i}}_{{x}_{2}}, {{C}_{i}}_{{x}_{3}}, \dots , {{C}_{i}}_{{x}_{n}}\right)$, and the Euclidean distance ${d}_{i}(i=1, 2, 3, \dots ,K)$ is calculated from ${C}_{i}$ to $\overline{M }$:
  5 $$d_{i} = \sqrt {\left( {A _{{x_{1} }} - C_{{ix_{1} }} } \right)^{2} + \ldots + \left( {A _{{x_{n} }} - C_{{ix_{n} }} } \right)^{2} } .$$

(4) Calculating the number of samples to be inserted in each cluster
  Let the number of samples to be inserted in K sub-clusters be ${n}_{1}, {n}_{2},{ n}_{3}, \dots ,{n}_{K}$, which satisfies $\sum_{i=1}^{i=K}{n}_{i}={l}_{add}$.
(5) The weight values assigned to each cluster is calculated. The sub-clusters with a shorter distance from class M, which are closer to the boundary, carry more useful information; thus, they receive higher weight values and allocate more synthetic samples. The sub-clusters with a longer distance from class M, which are further from the boundary, carry less useful information; thus, they will receive less weight values and allocate less synthetic samples. According to the above rules, the established formula to calculate the weight value W is
  6 $$W_{i} = 1 - \frac{{d_{i} }}{{\mathop \sum \nolimits_{i = 1}^{i = K} d_{i} }}.$$
  In the formula,$i=1, 2, 3, \dots ,K$, and ${W}_{i}$ denotes the weight value obtained for the i-th sub-cluster, which is given by the formula $\sum_{i=1}^{i=K}{W}_{i}=1$.
(6) The number of samples to be inserted in each cluster is calculated based on weight values. The number of samples to be inserted in the i-th sub-cluster ${n}_{i}$ is
  7 $$n_{i} = W_{i} \cdot l_{add} .$$
(7) Linear interpolation to synthesise a new sample Specific steps are as follows:
(8) Select a subcluster, randomly select one of the samples, and the k-nearest neighbour samples of this sample are determined based on the K-nearest neighbour algorithm. Let the number of new samples already synthesized by the current subcluster* i* be* r*_*i*_, the initial value of * r*_*i*_ is 0;
(9) If $k\ge \left({n}_{i}-{r}_{i}\right)$, A total of $\left({n}_{i}-{r}_{i}\right)$ new samples are generated via random linear interpolation between the sample and its $\left({n}_{i}-{r}_{i}\right)$ neighbours separately. The new sample synthesis of the current subcluster is completed, skip to step (5.5);
(10) If $k<\left({n}_{i}-{r}_{i}\right)$, A total of k new samples are generated via random linear interpolation between the sample and its k neighbours separately. Update the value of ${r}_{i}$;
(11) Randomly select one of the remaining samples of the current sub-cluster $i$, repeat steps (5.2) and (5.3);
(12) Repeat the above steps to complete the synthesis of new samples for other clusters.
  In the sub-cluster, a random sample selected based on the K nearest neighbors is ${x}_{k1}$, then the synthesized new sample ${x}_{new}$ is
  8 $$x_{new} = x + rand\left( {0,1} \right) \times \left( {x - x_{k1} } \right)$$

The new samples generated by all subclusters are added to the original training sample S, and a balanced dataset ${S}^{^{\prime}}$ with a balance rate of ${E}^{^{\prime}}$ is finally obtained.

**Figure 2:**
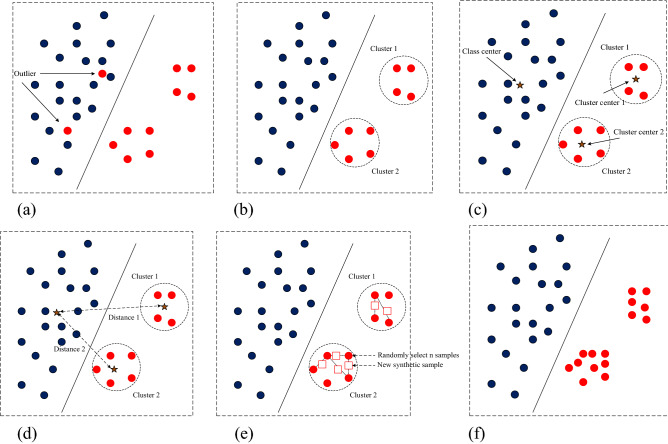
Simulation process of SD-KMSMOTE method. (**a**) Original dataset; (**b**) Filtering isolated sample points and generating clusters; (**c**) Calculating the majority class centres and the cluster centres of each cluster of the minority class; (**d**) Calculating the distance of each cluster to class M; (**e**) Synthesizing new samples; (**f**) The obtained balanced dataset.

The simulation process of the SD-KMSMOTE method proposed in this study is shown in Fig. 2. The simulated dataset on the two-dimensional plane has a total of two classes; blue dots represent the majority class, and red dots the minority class. Figure 2a shows the original distribution of the dataset as well as the isolated points, and Fig. 2b shows the clusters generated using the k-means method after removing the noise. Figure 2c shows the class centres of the majority class samples and the cluster centres of each cluster, and Fig. 2d shows the distance from each cluster to the centre of the class. Based on the distance, the weight is calculated according to Eq. 6, and the number of new samples synthesised by each cluster is allocated. These new samples are represented by the hollow squares in Fig. 2e. Finally, the balanced dataset obtained using the SD-KMSMOTE method is shown in Fig. 2f.

**Figure 3:**
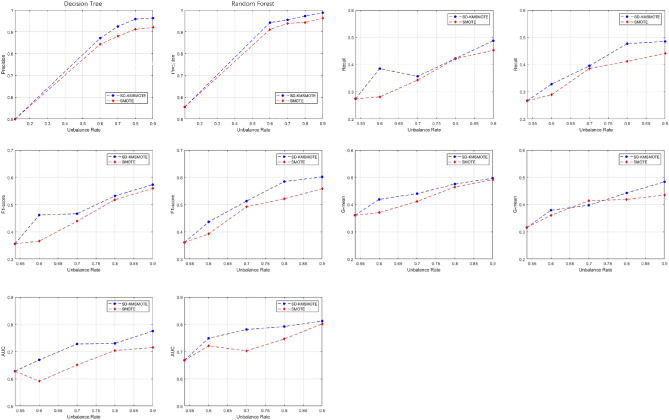
Comparison of evaluation indexes of **Pima** under different equilibrium rates.

## Experiment

In this section, the performance of the proposed SD-KMSMOTE oversampling method is demonstrated. We selected several classic over-sampling methods, including SMOTE, Bordline-SMOTE1^26^, Borderline-SMOTE2^26^ , and ADASYN^30^, as comparison methods, and explained and compared them in two parts: evaluation metrics and the analysis of experimental results. It is proved that the method proposed in this study is superior for imbalanced data processing and learning.

### Evaluation metric

Generally, we evaluate the good performance of an oversampling method by combining the classification algorithms. However, owing to the nature of the imbalanced classification problem, the accuracy and error rates that are generally used to evaluate classifier performance may no longer be applicable. This is because in an imbalanced classification problem, we are primarily concerned with whether minority classes are correctly classified; however, the accuracy of the overall performance in the classifier basically depends on the number of majority classes that are correctly classified. If all the majority classes are correctly classified and all the minority classes are incorrectly classified, the classifier can still easily achieve a high accuracy rate, but in reality, the classifier is ineffective.

Therefore, for the imbalanced data problem, this study selects some representative metrics to evaluate the performance of the imbalanced data to assess the classification results. These metrics include precision, recall, F1-score, G-mean, and the AUC. In the binary classification problem, we consider the categories with small numbers as positive examples and those with large numbers as negative examples. Table 1 shows the confusion matrix for the binary classification problem, and the formulas for each indicator obtained according to Table 1 are as follows:

9 $$Precision = \frac{TP}{{TP + FP}}$$

10 $$call = \frac{TP}{{TP + FN}}$$

11 $$Specificity = \frac{TN}{{TN + FP}}$$

12 $$F1 - score = 2 \cdot \frac{Precision \cdot Recall}{{Precision + Recall}}$$

13 $$G - mean = \sqrt {Recall \cdot Specificity}$$

14 $$FPR = \frac{FP}{{TN + FP}}$$

**Table 1:** Confusion matrix of binary classification problem.

	Predicted positive	Predicted negative
Actually positive	True positive (TP)	False positive (FP)
Actually negative	True negative (TN)	False negative (FN)

The horizontal coordinate of the ROC curve is the false positive rate (FPR), the vertical coordinate is the true positive rate (TPR), and the AUC is the area under the ROC curve.

### Part1 experimental results

We first use the method proposed in this paper to synthesise the new dataset by setting different balance rates using seven imbalanced datasets from the Kaggle. The basic information of the seven datasets used in this study is presented in Table 2.

**Table 2:** The description of the data sets.

The name of data set	Sample size	Number of attributes	Target (minority)	Equilibrium rate(E)	Percentage of minority class
Pima	768	8	1	53.6%	24.90%
Haberman	306	3	die	36.0%	26.47%
*E. coli*	336	7	imU	11.62%	10.42%
Wine	178	13	3	36.92%	26.97%
Breastcancer	699	10	4	52.62%	34.48%
SPECT heart	267	22	0	25.94%	20.6%
SPECTF heart	267	44	0	25.94%	20.6%

The experimental environments in this section were all conducted in MATLAB 2020 (MathWorks). For the method described in this study, it is necessary to perform clustering and calculate the number of samples inserted in each cluster before synthesising new samples. For this purpose, Table 3 lists some of the parameter values calculated using the method in this study before synthesising new samples.

**Table 3:** The parameter information of SD-KMSMOTE.

The name of data set	Number (minority)	Samples cluster1	Samples cluster2	*D*_1_	*D*_2_	*W*_1_	W_2_
Pima	268	182	86	51.927	196.66	0.7911	0.2089
Haberman	81	26	55	13.950	6.923	0.3317	0.6683
*E. coli*	35	16	19	0.4430	0.5340	0.5466	0.4536
wine	48	24	24	252.448	68.206	0.2127	0.7873
Breastcancer	241	108	133	10.368	16.137	0.6088	0.3912
SPECT heart	55	15	40	1.169	1.462	0.5556	0.4444
SPECTF heart	55	38	17	38.800	40.324	0.5096	0.4904

As shown in Table 3, we set the number of clusters of each dataset to two, and calculate the weight value of each cluster (${W}_{1}$ and ${W}_{2}$) according to the calculated Euclidean distance from each cluster centre to the centre of the majority classes (${D}_{1}$ and ${D}_{2}$). According to the weight value, the number of samples to be synthesised for each cluster at different balance rates is calculated.

### Part2 experimental results

**T**o verify the performance of the proposed method in this study with datasets of different balancing rates, SMOTE is introduced as a comparison group in this section, and the first three datasets (Pima, Haberman, *E. coli*) are selected for experiments. After obtaining the synthetic dataset at different equilibrium rates using the methods of this study and SMOTE, we use decision trees and random forests as classifiers to obtain the classification results for the original and synthetic datasets at different equilibrium rates. By comparing the original dataset with the evaluation metrics under different balance rates, it is proved that it is necessary and effective to balance the imbalanced dataset.

MATLAB 2020 is still implemented as the experimental environment for this part of the study. To make a fair comparison, the parameters of the classification algorithm are set as the default values. In addition, we divided each data set into 10 groups on average for a total of 30 experiments. Each time, 3 subsets of the data set were randomly selected as the test set and the remaining 7 subsets were selected as the training set. Finally, the average of five evaluation indexes obtained from 30 experiments was selected as the result of performance comparison of different classifiers. The number of samples from each of the three datasets used for training and testing is shown in Table 4.

**Table 4:** The number of samples used in the experiment.

E	Number of Pima			Number of haberman			Number of *E. coli*		
	Train	Test	Total	Train	Test	Total	Train	Test	Total
original	537	231	768	214	92	306	235	101	336
0.6	560	240	800	252	108	360	337	144	481
0.7	595	255	850	267	115	382	358	153	511
0.8	630	270	900	283	122	405	379	163	542
0.9	665	285	950	299	128	427	400	171	571

In the classification experiments, we use decision trees and random forests to represent single and combined classifiers, respectively, to verify the performance of the proposed method.

**Figure 4:**
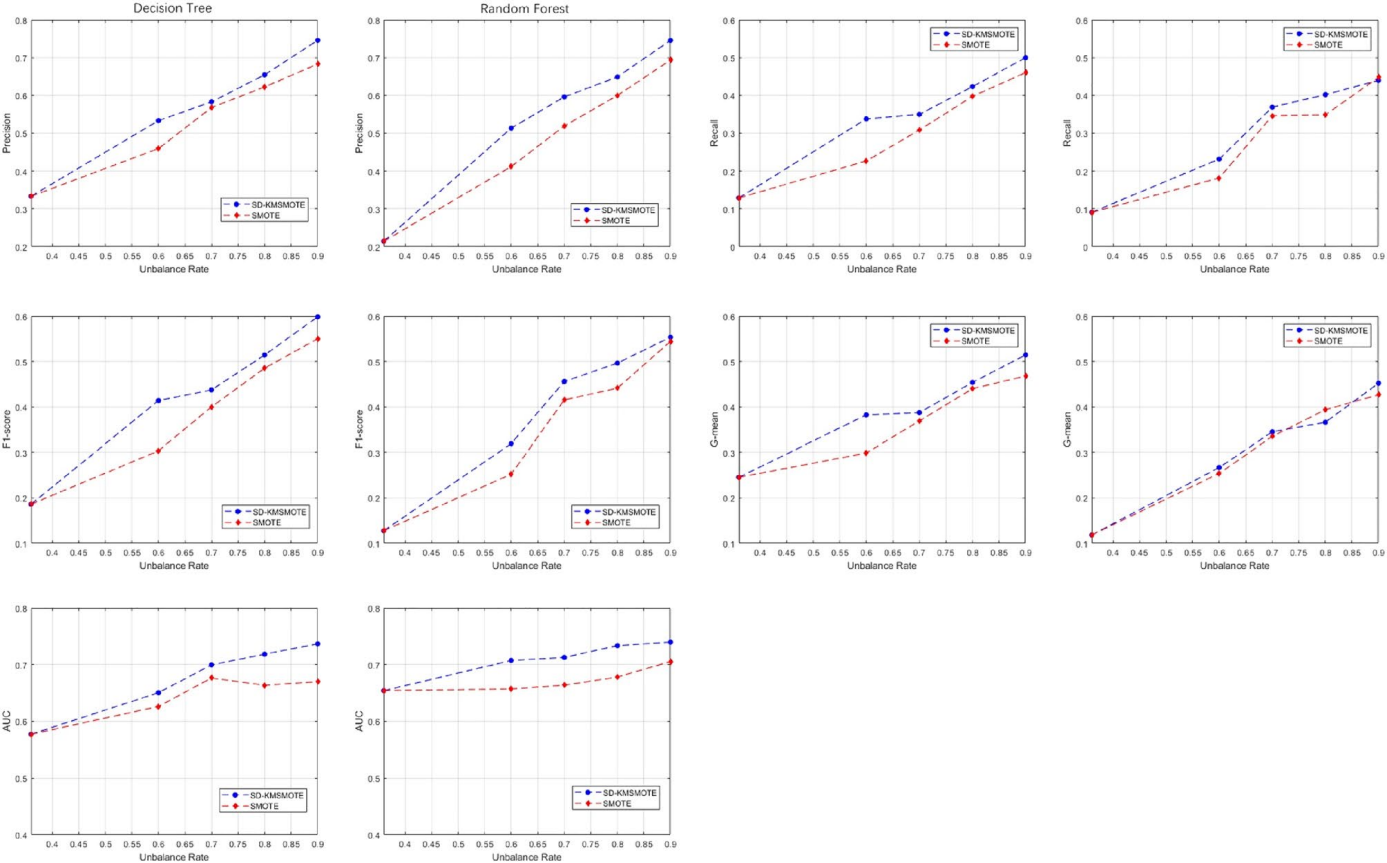
Comparison of evaluation indexes of **Haberman** under different equilibrium rates.

**Figure 5:**
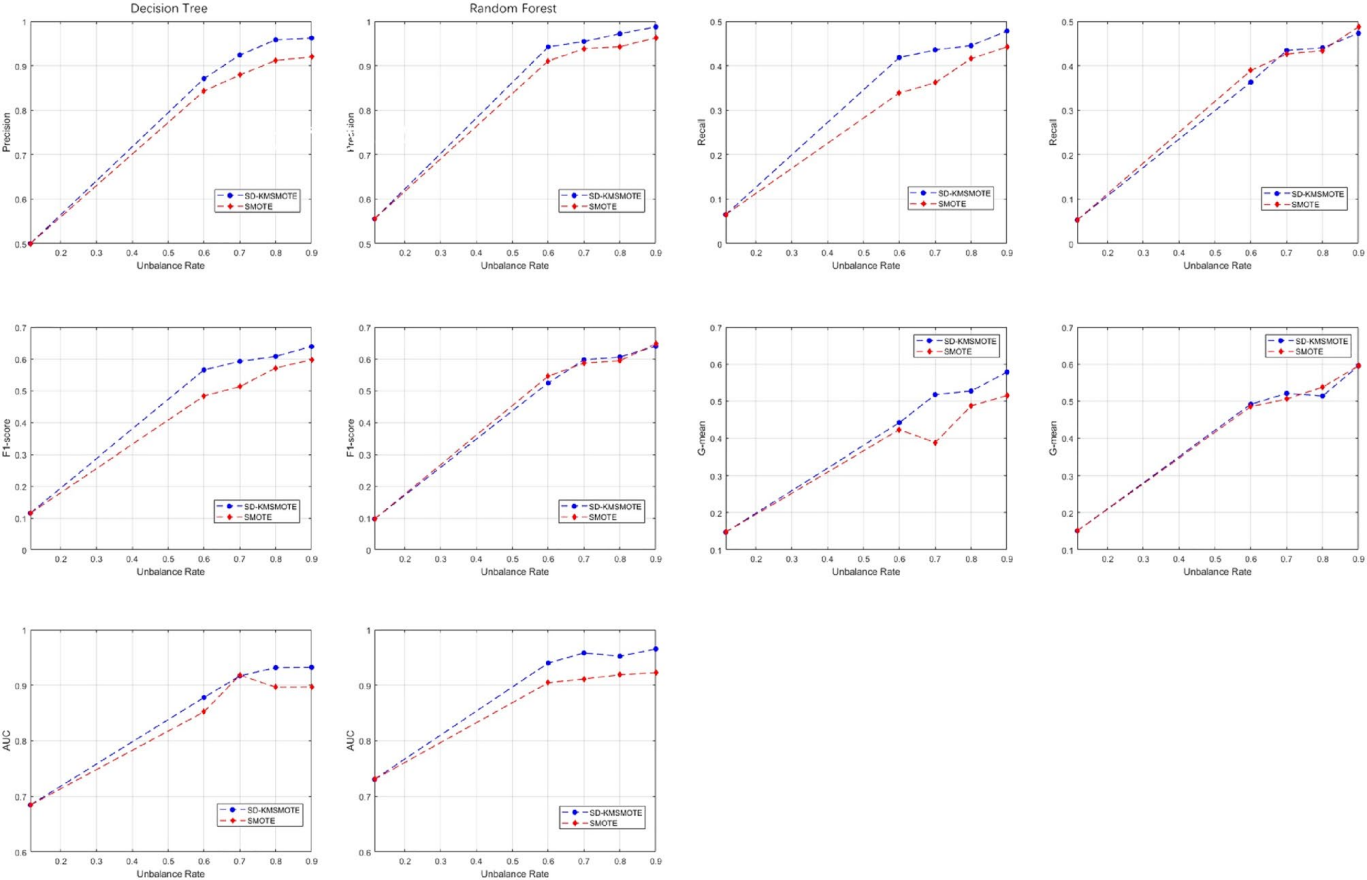
Comparison of evaluation indexes of ***E.coli*** under different equilibrium rates.

Figures 3, 4, 5 show the comparison of five evaluation metrics obtained using decision tree and random forest classification algorithms for three synthetic datasets with different equilibrium rates obtained using SD-KMSMOTE and SMOTE. We can observe that, the improvement of each index is clear compared with the original imbalanced dataset, and the overall trend of each index also increases as the balancing rate increases, especially for the dataset with a lower original balancing rate. This illustrates that it is necessary to balance the imbalanced datasets before classifying them; therefore, our proposed method is meaningful. However, comparing the experimental results of the synthetic datasets obtained using the two classification algorithms to process SMOTE, our proposed SD-KMSMOTE method obtained five evaluation metrics, namely precision, recall, F1 score, G-mean, and AUC, at four different balance rates for the three datasets, which is a total of 120 comparison results. From Figs. 3, 4, 5, we can see that among the 120 results, the performance of the evaluation metrics of SMOTE is better than that of SD-KMSMOTE in only nine results. Eight of these nine results are of the random forest algorithm, and five of them are from the Ecoi dataset. Thus, the result may be related to the low balance rate of the original dataset and the performance of the classifier itself. In conclusion, through comparative experiments, we demonstrate the effectiveness of the SD-KMSMOTE method proposed in this study in dealing with imbalance learning. The overall performance of the synthetic datasets sampled using SD-KMSMOTE at different equilibrium rates is significantly better than that of the datasets obtained using the traditional SMOTE method.

### Part3 experimental results

To validate the performance of the proposed SD-KMSMOTE method, we select four classical algorithms, namely borderline-SMOTE1^26^, borderline-SMOTE2^26^, ADASYN^30^, and SMOTE, for further comparison. We use the seven imbalanced datasets in Table 2 for binary classification. We uniformly choose a balance rate of 0.9, synthesise the new dataset, and obtain the classification results using decision trees and random forests as classifiers to calculate five evaluation metrics to fully verify the superiority of SD-KMSMOTE.

**Figure 6:**
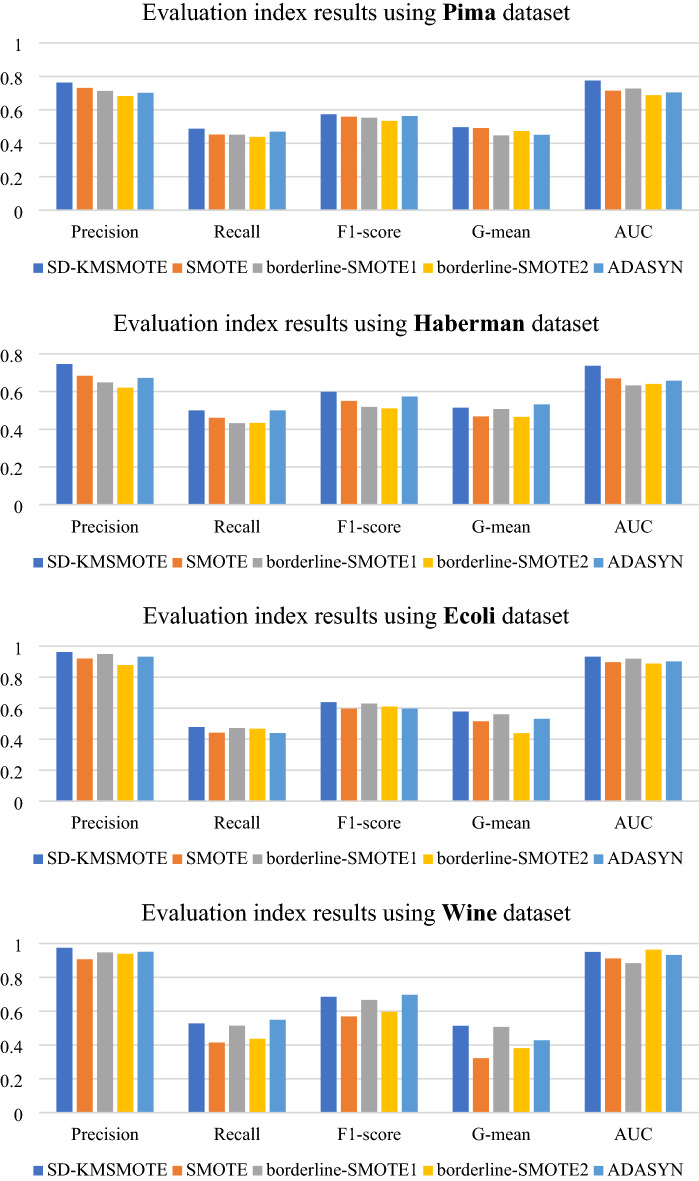

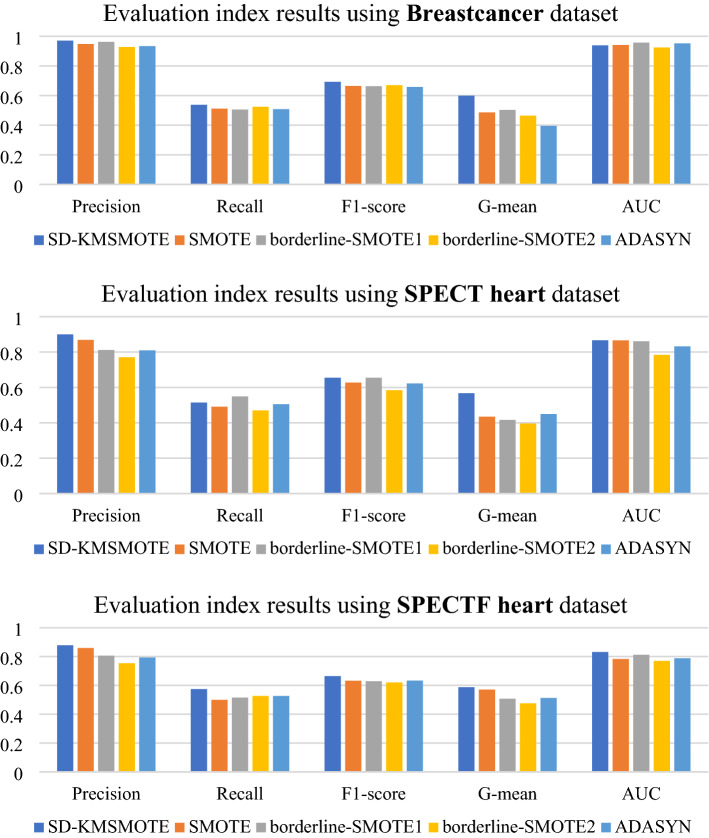
Evaluation indexes of oversampling methods using Decision Tree.

**Figure 7:**
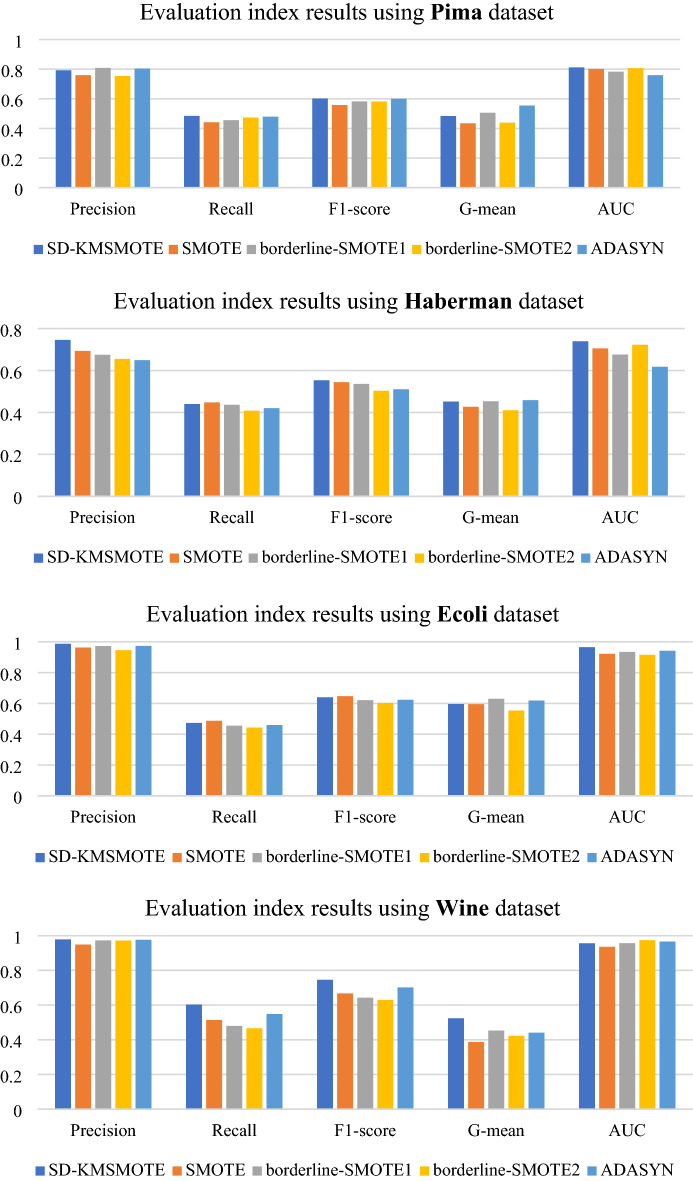

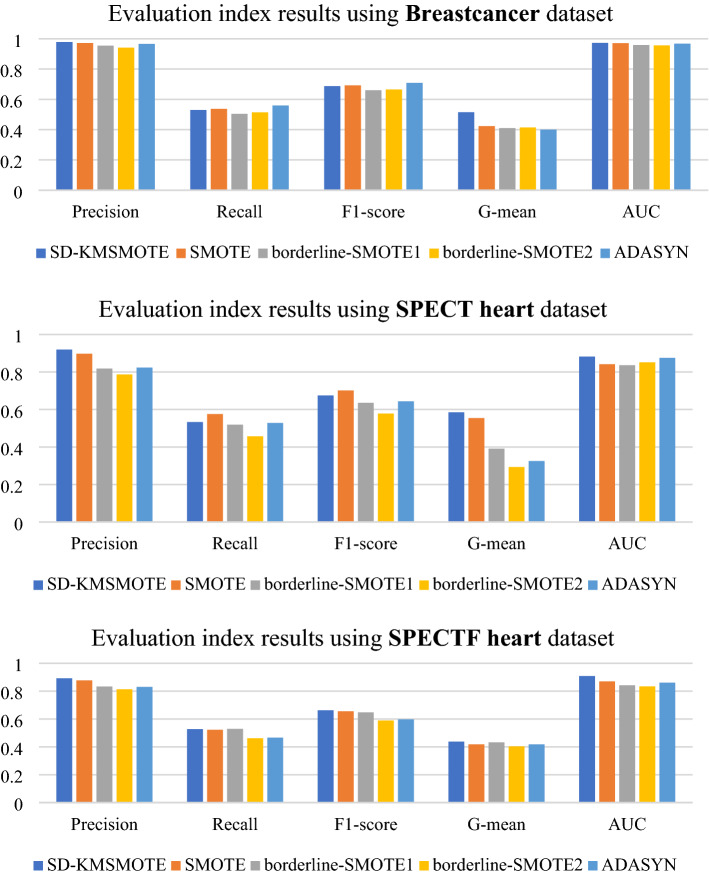
Evaluation indexes of oversampling methods using Random Forest.

In this part of the experiments, we use Borderline-SMOTE1, Borderline-SMOTE2, and ADASYN methods from Python's imblearn library to obtain a synthetic dataset with a balance rate of 0.9. The remainder of the experimental environment is still performed using MATLAB 2020 with the same specific parameter settings and other instructions as in 4.2.1. Figures 6, 7 show the comparison results of borderline-SMOTE1, borderline-SMOTE2, ADASYN, SMOTE, and the proposed SD-KMSMOTE method.

Figures 6, 7 show the comparative ranking of the five evaluation metrics obtained by the five oversampling methods combined with the two classification algorithms. We count 70 ranking situations, the result is shown in Table 5. It can be observed that our proposed SD-KMSMOTE method achieves the best performance in 51 of the 70 cases. Moreover, we can also observe that SD-KMSMOTE always ranks ahead in the performance of the AUC value. This may be because SD-KMSMOTE comprehensively exploits the useful information in each region of minority class samples and better improves the classifier's ability for positive and negative cases, thereby obtaining more desirable AUC values. By comparing the performances of different sampling methods, it is demonstrated that SD-KMSMOTE has the best performance among the five methods. In addition, it can be observed that SD-KMSMOTE is not in the top three only three times in all 70 cases; when SD-KMSMOTE ranks 2nd or 3rd, the difference between its values and the 1st or top two values in minimal, which proves that SD-KMSMOTE has good stability and further validates the superiority of our proposed SD-KMSMOTE method.

**Table5:** Heat map table of experimental results.

Method	Rank 1	Rank 2	Rank 3	Rank 4	Rank5
SD-KMSMOTE	51	11	5	3	0
SMOTE	5	20	19	15	11
borderline-SMOTE1	5	14	19	24	8
borderline-SMOTE2	3	4	5	14	44
ADASYN	7	20	22	15	6

## Conclusion

The current expansion in the field of artificial intelligence has led to an imbalanced learning problem that is still receiving increasing research attention. Unlike most existing oversampling methods that randomly sample or sample only for a particular region, this study proposes an imbalanced data oversampling method, SD-KMSMOTE, based on the spatial distribution of minority class samples. In this study, we focus on the spatial distribution of minority class samples, allocate more synthetic new samples in the boundary region, allocate a particular number of new samples to be generated in the safe region, and improve the performance of the imbalanced learning problem in three main ways. (1) Adding filtering noise preprocessing. Before the new sample synthesis operation, the isolated minority class sample points, where the surrounding samples all belong to the majority class samples, are screened as noise. (2) K sub-clusters are generated for a small number of sample classes using the k-means method. The cluster is taken as a unit, and the sub-clusters that contain more useful information are assigned larger weight values and a greater number of synthesised samples. (3) Calculation rules are constructed for weight values. By calculating the Euclidean distance from the cluster centre of each cluster to the class centre of the majority class sample, the clusters that are closer to the majority class sample are assigned a higher weight value. In this study, five evaluation metrics that are commonly used in imbalanced learning are utilized, and two experiments are conducted to analyse the method’s performance under different balance rates and the performance of different sampling methods. The experimental results show the effectiveness, stability, and superiority of the proposed method in dealing with imbalanced learning.

In the future, our research will focus on two aspects. The dataset selected for this experiment has some noisy data, which has not been elaborated on. Therefore, more noise will be subsequently introduced to address noisy samples in imbalanced data using the method presented in this study. Furthermore, the method presented in this study will be extended to the study of multiclassification imbalance learning.

## Data Availability

The data used to support the results of this study is publicly available and can be obtained from the website http://archive.ics.uci.edu/ml/.
